# Characterization of hydrocarbon-degrading and biosurfactant-producing ***Pseudomonas*** sp. P-1 strain as a potential tool for bioremediation of petroleum-contaminated soil

**DOI:** 10.1007/s11356-014-2872-1

**Published:** 2014-04-18

**Authors:** Magdalena Pacwa-Płociniczak, Grażyna Anna Płaza, Anna Poliwoda, Zofia Piotrowska-Seget

**Affiliations:** 1 Department of Microbiology, University of Silesia, Jagiellońska 28, 40-032 Katowice, Poland; 2 Environmental Microbiology Department, Institute for Ecology of Industrial Areas, Kossutha 6, 40-844 Katowice, Poland; 3 Faculty of Chemistry, Opole University, Pl. Kopernika 11, 45-040 Opole, Poland

**Keywords:** Hydrocarbon degradation, Biosurfactant production, *Pseudomonas* sp. P-1, Crude oil, Rhamnolipid, *rhl* gene

## Abstract

The *Pseudomonas* sp. P-1 strain, isolated from heavily petroleum hydrocarbon-contaminated soil, was investigated for its capability to degrade hydrocarbons and produce a biosurfactant. The strain degraded crude oil, fractions A5 and P3 of crude oil, and hexadecane (27, 39, 27 and 13 % of hydrocarbons added to culture medium were degraded, respectively) but had no ability to degrade phenanthrene. Additionally, the presence of gene-encoding enzymes responsible for the degradation of alkanes and naphthalene in the genome of the P-1 strain was reported. Positive results of blood agar and methylene blue agar tests, as well as the presence of gene *rhl*, involved in the biosynthesis of rhamnolipid, confirmed the ability of P-1 for synthesis of glycolipid biosurfactant. ^1^H and ^13^C nuclear magnetic resonance, Fourier transform infrared spectrum and mass spectrum analyses indicated that the extracted biosurfactant was affiliated with rhamnolipid. The results of this study indicate that the P-1 and/or biosurfactant produced by this strain have the potential to be used in bioremediation of hydrocarbon-contaminated soils.

## Introduction

Contamination of soil by petroleum hydrocarbons and their derivatives is a serious environmental problem all over the world. Among a variety of the remediation methods, bioaugmentation, involving the introduction of microorganisms into contaminated water or soil, is widely used for cleaning up environments polluted with organic compounds (Chang et al. 2011; Federici et al. 2012), co-contaminated with hydrocarbons and heavy metals (Alisi et al. 2009), or polluted with heavy metals (Płociniczak et al. 2013).

In bioaugmentation of petroleum-contaminated soil, hydrocarbon-degrading bacteria are applied to polluted environments in order to accelerate the degradation of toxic compounds (Margesin and Schinner 2001). One of the main problems that limit the effectiveness of this process is very low solubility and high hydrophobicity of oil pollutants. These compounds strongly bind to soil particles, and thereby they are poorly available for bacterial cells (Johnsen et al. 2005). Solution to this problem is the use of biosurfactants or biosurfactant-producing microorganisms. Biosurfactants are a structurally diverse group of surface-active substances produced by microorganisms that exhibit the ability to reduce surface and interfacial tension. They may enhance hydrocarbon bioremediation by two mechanisms. The first includes the increase the substrate availability for microorganisms, while the second involves interaction with the cell surface, which increases the hydrophobicity of the surface, allowing hydrophobic substrates to associate more easily with bacterial cells (Pacwa-Płociniczak et al. 2011).

The application of biosurfactants for large scale is limited by the high costs of their production. These costs may be reduced by the use of agro-industrial wastes to culture biosurfactant-producing bacteria (Płaza et al. 2011). Therefore, in our preliminary studies, the ability of P-1 to grow on various agro-industrial substrates has been tested. Among 21 tested media, molasses appeared to be the most suitable medium for culturing these bacteria (Pacwa-Płociniczak et al, Evaluation the usefulness of *Pseudomonas aeruginosa* P-1 strain for bioremediation of petroleum-polluted soils, Personal Communication).

The aims of the study were to (1) characterize the ability of the *Pseudomonas* sp. P-1 strain to degrade hydrocarbons and produce biosurfactants when grown on molasses, and (2) to characterize the structure of the biosurfactant produced by P-1.

## Materials and methods

### Bacterial strain—isolation and detection of biosurfactant production

Bacterial strain P-1 used in this study was isolated using an enrichment technique from petroleum-polluted soil taken from the area around of a 100-year-old oil refinery in Czechowice-Dziedzice, Upper Silesia, Poland, as described by Berry et al. (2006) and Płaza et al. (2006). Expedite methods were used to detect the ability of P-1 for biosurfactant production. Hemolytic activity was carried out as described by Carrillo et al. (1996). Strain P-1 was streaked onto blood agar plates containing 40 g of blood agar base (Becton Dickinson, Sparks, MD) and 50 ml of sheep blood (BIOMED-LUBLIN) per litre. Plates were incubated for 48 h at 28 °C. The plates were inspected for zones of clearing around the colonies, indicative of biosurfactant production. The synthesis of extracellular glycolipid biosurfactant was detected using the Siegmund and Wagner (1991) technique. A spot of culture grown on LB medium was placed on a methylene blue agar plate (0.2 g of cetyltrimethylammonium bromide (CTAB), 0.005 g of methylene blue, 20 g of glucose, 0.7 g of KH_2_PO_4_, 0.9 g of Na_2_HPO_4_, 2 g of NaNO_3_, 0.4 g of MgSO_4_ × 7H_2_O, 0.1 of CaCl_2_ × 2H_2_O, 20 g of agar and 2 ml of a trace elements solution containing 2 g of FeSO4 × 7H_2_O, 1.5 g of MnSO_4_ × H_2_O and 0.6 g of (NH_4_)_6_Mo_7_O_24_ × 4H_2_O per litre of deionized water). After 48 h of incubation at 28 °C, the plate was observed for the formation of a dark blue halo around the culture spot, indicating the formation of an insoluble ion pair between the anionic glycolipid biosurfactant and the cationic CTAB–methylene blue agar complex.

### Identification of isolate

Strain P-1 was identified on the basis of 16S rRNA gene sequence analysis. For 16S rRNA gene amplification, the universal bacterial primers 8 F (5′ AGTTTGATCATCGCTCAG 3′) and 1492R (5′ GGTTACCTTGTTACGACTT 3′) targeting fragment size 1,484 bp were used (Lonergan et al. 1996). The PCR was run with a mixture containing 1 μl of the DNA template, 0.2 μM of each primer, 10× reaction buffer (Fermentas), 1.5 mM of MgCl_2_ (Fermentas), 200 μM of dNTP and 1 U of DreamTaq DNA polymerase (Fermentas) in a C1000 Touch™ Thermal Cycler (BioRad). PCR amplification was performed at 94 °C for 5 min, 3 cycles at 94 °C for 45 s, 57 °C for 30 s, 72 °C for 120 s; 3 cycles at 94 °C for 45 s, 56 °C for 30 s, 72 °C for 120 s; 3 cycles at 94 °C for 45 s, 55 °C for 30 s, 72 °C for 120 s; 26 cycles at 94 °C for 45 s, 53 °C for 30 s, 72 °C for 120 s; and a final elongation cycle at 72 °C for 5 min. Gene sequencing was performed by using the Big Dye® Terminator Cycle Sequencing Kit (Applied Biosystem) and the AbiPrism®3100 Genetic Analyzer. The obtained sequences were compared to known 16S rRNA gene sequences using the BLAST server at the National Center for Biotechnology Information (NCBI; http://www.ncbi.nlm.nih.gov/). DNA sequences were aligned using CLUSTAL W. Phylogenetic analyses were performed by the neighbour-joining (NJ) method testing the support for the phylogeny with a bootstrap analysis based on 1,000 replicates using the MEGA ver. 6.0.

### Detection of genes encoding enzymes involved in rhamnolipid synthesis

The primers rhlABF 5′ CAG GCC GAT GAA GGG AAA TA 3′ and rhlABR 5′ AGG ACG ACG AGG TGG AAA TC 3′ (Kumar et al. 2008) targeting fragment size 777 bp were used to detect potential rhamnolipid synthesis by the P-1 strain. The PCR was run with a mixture containing 1 μl of the DNA template, 0.2 μM of each primer, 10× reaction buffer (Fermentas), 1.5 mM of MgCl_2_ (Fermentas), 200 μM of dNTP and 1 U of Taq DNA polymerase (Fermentas) in a C1000 Touch™ Thermal Cycler (BioRad). PCR amplification was performed at 95 °C for 5 min, and 30 cycles of 30 s at 95 °C, followed by annealing for 1 min at 50 °C and an extension step of 2 min at 72 °C and a final extension step of 10 min at 72 °C. The experiment included a control reaction mixture without added DNA.

### Detection of gene encoding enzymes involved in hydrocarbon degradation

The primers alkBfd 5′ AAC TAC MTC GAR CAY TAC GG 3′ and alkBRd 5′ TGA MGA TGT GGT YRC TGT TCC 3′ (where M = AC, R = AG and Y = CT) (Powell et al. 2006) and nahAc-7 F 5′ ACT TGG TTC CGG AGT TGA TG 3′ and nahAc-7R 5′ CAG GTC AGC ATG CTG TTG TT 3′ (Park and Crowley 2006) targeting fragments 100 and 136 bp were used to detect alkane monooxygenase and naphthalene dioxygenase genes in the P-1 strain. The PCR was run with a mixture containing 1 μl of the DNA template, 0.2 μM of each primer, 10× reaction buffer (Fermentas), 1.5 mM of MgCl_2_ (Fermentas), 200 μM of dNTP and 1 U of DreamTaq DNA polymerase (Fermentas) in a C1000 Touch™ Thermal Cycler (BioRad). PCR amplification was performed at 95 °C for 5 min, and 35 cycles of 1 min at 94 °C followed by annealing for 30 s at 49 °C and an extension step of 45 s at 72 °C for the *alkB* gene and 37 cycles of 20 s at 94 °C followed by annealing for 15 s at 56 °C and an extension step of 15 s at 72 °C for the *nahAc* gene, and then a final extension step of 5 min at 72 °C. The experiment included a positive control with DNA isolated from the strain with high ability to degrade aromatic hydrocarbons (*Arthrobacter* sp. F1A strain, Markowicz and Piotrowska-Seget, Isolation and characterization of bacterial strains capable of degradation of phenanthrene, Personal Communication) for the *nahAc* gene and a control reaction mixture without added DNA for both genes.

### Growth of isolates on hydrocarbons and determination of hydrocarbon concentration

Replicate flasks containing 100 ml of M9 minimal salt medium (Viramontes-Ramos et al. 2010) with 100 μl of crude oil, its fraction A5, P3 and hexadecane and 20 mg of phenanthrene were prepared. A5 and P3 are distillation fractions of crude oil containing components of diesel oils and light fuel oils and components of high fuel oils (raw material to mazout), respectively (Płaza et al. 2008). The flasks were inoculated with 1 ml of the P-1 strain suspended in sterile saline. Non-inoculated flasks were prepared as controls. The cultures were grown aerobically at 28 °C for 28 days with constant shaking (120 rpm). After that, the residual hydrocarbon concentrations were determined. Crude oil and its fraction A5 and P3 were extracted from bacterial culture using the liquid–liquid extraction technique with hexane as an extraction solvent. The extract was cleaned up on a column filled with Florisil sorbent and evaporated to a volume of 1 ml. Mineral oil (in the range C_10_–C_40_) was determined by gas chromatography coupled with a flame ionization detector (GC/FID), according to the accredited procedure PB-16 (2009). Hexadecane concentration was determined according to the method described by Wypych and Mańko (2002), in which HS-SPME-GC/MS was used. The analysis was carried out with a Star 3400 Cx gas chromatograph (equipped with a ^63^Ni Electron Capture Detector); it was coupled to a mass spectrometer, Saturn 3 and Autosampler 8200 Cx (Varian) with 10 ml autosampler vials. The chromatographic column with the phase DB624 and length 30 m × 0.32 mm ID (1.8 μm film thickness) was used. Helium was used as the carrier gas: purity 99.999 %, flow capacity of 1.0 ml min^−1^ (in a temperature of 35 °C). The gas chromatographic conditions were as follows: the oven temperature was held at 40 °C for 10 min, then increased to 250 °C at 10 °C min^−1^, and finally increased at 5 °C min^−1^ to 270 °C. The total analysis time was 45 min. The injector temperature was 250 °C. The MS operating conditions were the following: the mass range scanned was 30–250 amu at 1 s/scan, the temperature of the ion trap was 170 °C, multiplier voltage was 2,700 V and ionization energy was 70 eV (electron impact mode EI). The transfer line temperature was 250 °C. The temperature of the ECD detector (^63^Ni) was 300 °C. Three types of SPME fibre were used (Supelco, Bellefonte) in an autosampler set of the following stationary phases: 100 μm polydimethylosiloxane, 7 μm polydimethylosiloxane and 85 μm polyacrylate. The SPME fibre was conditioned prior to use in order to reduce bleeding by heating in a split/splitless injector with an open purger, in a helium stream: fibre 100 μm PDMS at a temperature of 250 °C (for 1 h), fibre 7 μm PDMS at a temperature of 320 °C (for 2 h) and fibre 85 μm polyacrylate at a temperature of 300 °C (for 2 h). Phenanthrene was extracted using the solid phase extraction (spe) technique with a mixture of octadecyl and amino phase as a sorbent and eluted with dichloromethane. Then, the eluate was evaporated to a volume of 1 ml. Phenanthrene was determined by high-performance liquid chromatography coupled with a fluorescence detector (HPLC/FLD), according to accredited procedure PB-06 (2010). For each measurement, three replicates were used.

### Determination of rhamnolipid production and yield

The drop-collapse technique was carried out in the polystyrene lid of a 96-microwell plate (Biolog, Harward, CA, USA), as described by Płaza et al. (2006). One hundred microlitres of each supernatant was added to the wells of a 96-well microtitre plate lid, then 2 μl of crude oil was added to the surface of the culture supernatant, and the flattening of the oil drop indicating a positive result of the test was checked.

The oil-spreading technique was carried out according to Morikawa et al. (2000) and Youssef et al. (2004). Fifty millilitres of distilled water was added to Petri dishes, followed by the addition of 100 μl of crude oil to the surface of the water. Then, 10 μl of each culture supernatant was put onto the crude oil surface. The diameter of the clear zone on the oil surface was observed.

The emulsifying activity was determined using a modification of the method described by Cooper et al. (1987). To 2 ml of the bacterial cultures in a screw-cap tube, 3 ml of hydrocarbons (benzene, hexadecane, cyclohexane, diesel oil and A3 fraction of diesel oil) was added and vortexed at high speed for 2 min. The emulsion stability was determined after 24 h. The emulsification index (E24) was calculated as the percentage height of the emulsion layer to the total height of the liquid column.

Surface (ST) tension of the culture supernatants was determined with a Kruss Processor Tensiometer (model K12 Kruss, Germany) using the plate method. To increase the accuracy an average of triplicates was used for the study. All the assays were performed in triplicate with distilled water as the negative control and sodium dodecyl sulphate (SDS) as the positive control.

Rhamnolipid was quantified from the cell-free molasses culture as rhamnose (g l^−1^) using the phenol–sulphuric method (Dubois et al. 1956). One millilitre of the culture supernatant was mixed with 0.5 ml of 80 % phenol and 2.5 ml of concentrated sulphuric acid. After 10 min of incubation at room temperature, the absorbance was measured at 490 nm, and the rhamnose concentration was calculated using a standard curve prepared using different concentrations (0–4,000 mg l^−1^) of rhamnose. Rhamnolipid values were determined by multiplying rhamnose values by a coefficient of 3.4 obtained from the correlation (*y* = (0.0139*x* − 0.0058) × 0.68) of pure rhamnolipids/rhamnose (Benincasa et al. 2002). The experiment was carried out in triplicate.

### Extraction and structural analysis of biosurfactant

To get a biosurfactant for chemical analysis, the P-1 strain was grown in a molasses medium on a rotatory shaker (120 rpm) at 28 °C for 96 h. Then, the culture was centrifuged at 12,000 rpm for 30 min at 4 °C to remove the cells, and the biosurfactant was recovered from the supernatant. Its pH was adjusted to 2.0 with 1 N HCl and left overnight at 4 °C. The cloudy supernatant was twice extracted with an equal volume of chloroform/methanol (2:1) solution in a separating funnel. The pooled organic phase was evaporated under vacuum (Buchi, Germany). Then, the obtained brownish oily residue was characterized with spectroscopic techniques.

Electrospray ionization (ESI) mass spectra of analysed product were recorded on the LC-MS system consisting of high-performance liquid chromatography (Ultimate 3000, Dionex) with a diode-array detector and a high-resolution micrOTOF-QII (Bruker Daltonics, Germany) time-of-flight mass spectrometer. The chromatographic separation was performed on a Kinetex C18, 2.6 μm, 100 × 4.6 mm column. An acetonitrile/water gradient was used, starting with 25 % acetonitrile for 5 min, followed by a ramp of 25–100 % acetonitrile for 30 min. The ESI mass spectrum in the negative ion mode was acquired using a capillary voltage of −3.5 kV, and desolvatation gas (nitrogen) was heated to 150 °C. Full scan data were obtained by scanning from range *m*/*z* 50 to 1,000. The analysed sample of brownish oily product before LC-MS analysis was dissolved in methanol/water (1:1, *v*/*v*) and filtered (0.22 μm).

Infrared absorption spectra were recorded on an FTIR Nicolet Nexus spectrophotometer using KBr discs prepared as follows: 2 mg of brownish oily biosurfactant was ground with 600 mg of KBr and pressed with 15,000 kg for 30 s to obtain translucent pellets. All measurements consisted of 20 scans with a spectral resolution 2 cm^−1^.

The assignments of the glycolipid signals were carried out using ^1^H-NMR and ^13^C-NMR spectra with Bruker Ultrashield 400 MHz. The NMR spectra were determined in deuterated DMSO (dimethyl sulphate-d6), using tetramethyl silane (TMS) as the internal standard.

## Results

**Fig. 1:**
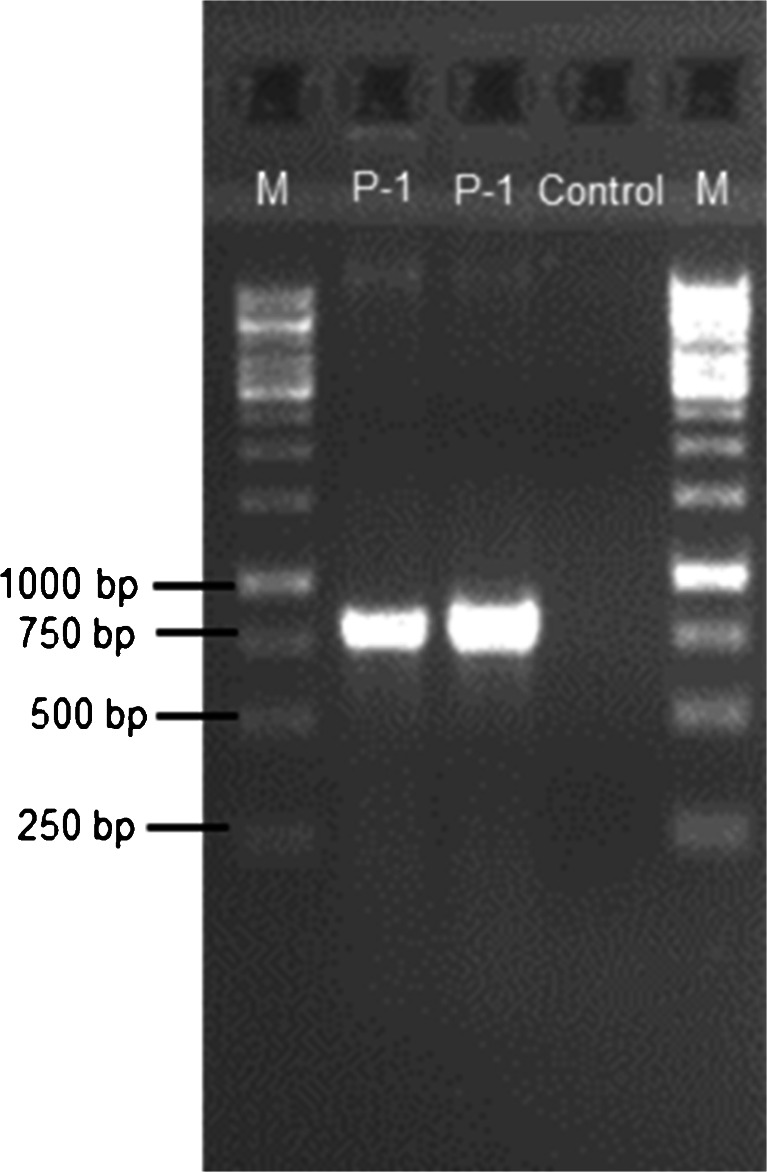
Agarose gel electrophoresis of PCR products of rhamnosyl transferase gene; *M*—1 kb DNA ladder; *P-1*—*Pseudomonas* sp. P-1 strain; *Control*—sample without DNA (1 % agarose gel)

The application of an enrichment technique for the isolation of bacteria from petroleum-polluted soil from the area of 100-year-old oil refinery in Czechowice-Dziedzice (Poland) enabled us to find several bacterial strains with the ability to degrade hydrocarbons and produce biosurfactants. However, only one strain (P-1) demonstrated a positive reaction for both blood agar and methylene blue agar tests, suggesting the potential ability of the isolate to synthesize glycolipid biosurfactant. This ability was confirmed by the polymerase chain reaction of the *rhl* gene. When the primers rhlABF and rhlABR were used, a single DNA fragment of the expected size (777 bp) was amplified in the P-1 strain, and no amplification was observed in the control without DNA (Fig. 1).

**Fig. 2:**
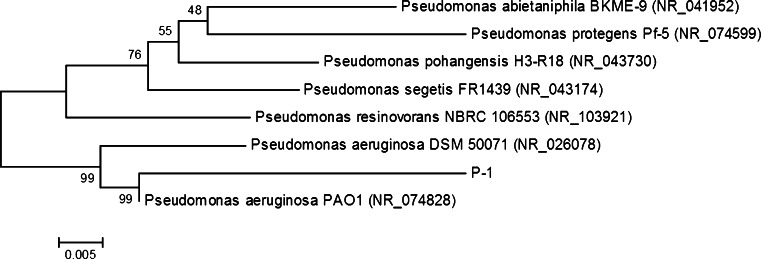
Neighbour-joining phylogenetic tree of bacteria based on 16S rRNA gene sequence comparisons. Bootstrap values are indicated at the branches from 1,000 replications. GenBank accession numbers are given *in parentheses*

The strain was identified using 16S rDNA gene sequence analysis as *Pseudomonas* sp. and designed as the P-1 strain. The phylogenetic analysis showed that the 16S rDNA sequence of P-1 had 99 % sequence similarity with strains *Pseudomonas aeruginosa* DSM 50071 and *P. aeruginosa* PAO1 (Fig. 2).

**Fig. 3:**
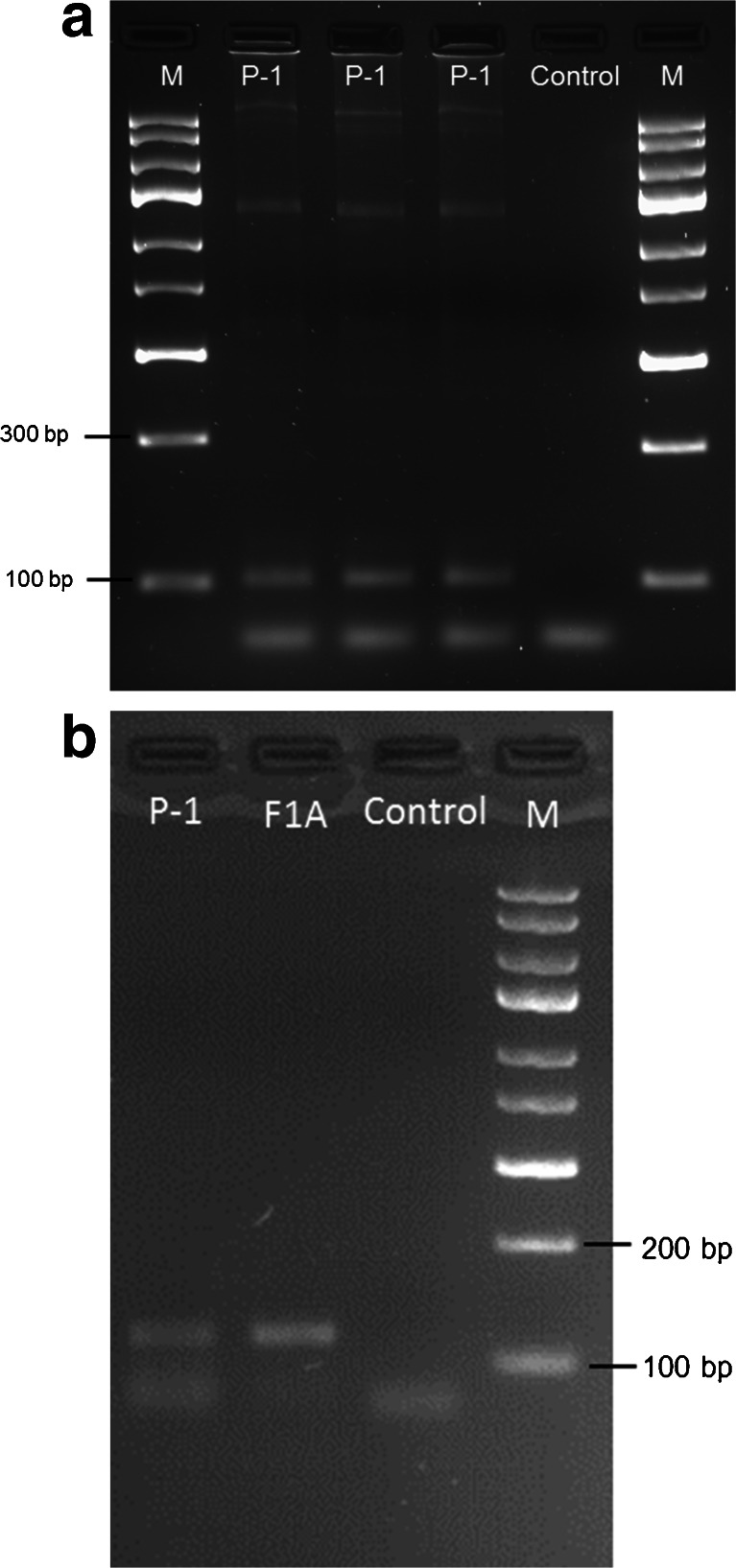
Agarose gel electrophoresis of PCR products of **a** alkane monooxygenase gene; *M*—express DNA ladder; *P-1*—*Pseudomonas* sp. P-1 strain; *Control*—sample without DNA (3 % agarose gel); **b** naphthalene dioxygenase gene; *M*—100 bp DNA ladder; *P-1*—*Pseudomonas* sp. P-1 strain; *F1A*—reference strain with high ability to degrade aromatic hydrocarbons (*Arthrobacter* sp., Markowicz and Piotrowska-Seget, Isolation and characterization of bacterial strains capable of degradation of phenanthrene, Personal Communication); *Control*—sample without DNA (2 % agarose gel)

**Fig. 4:**
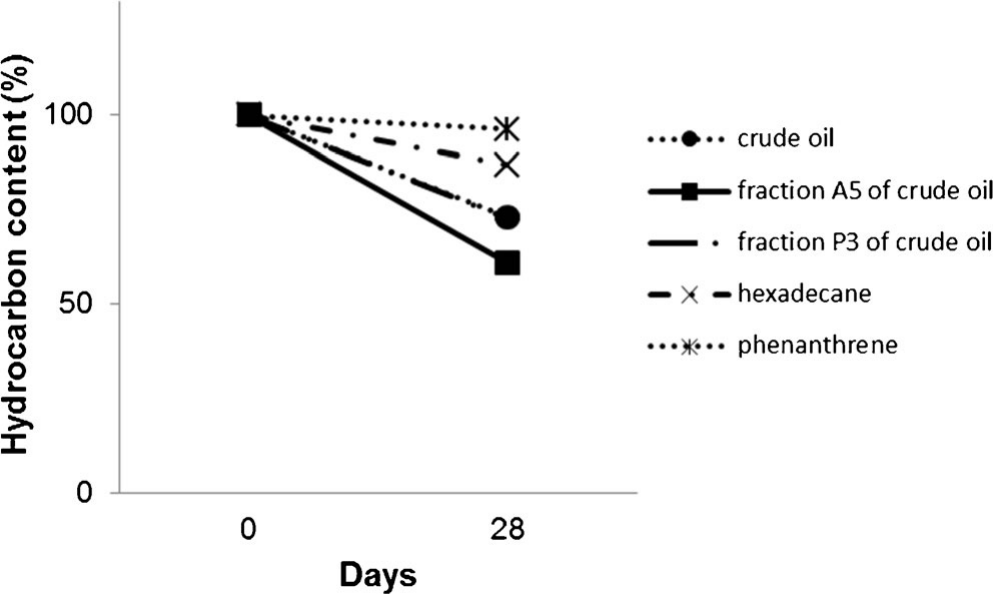
Degradation of hydrocarbons by P-1 strain

The use of the polymerase chain reaction and the sets of primers alkB and nahAc-7 confirmed the presence of genes involved in the degradation of alkanes and naphthalene in the genome of the P-1 strain. In the gel (Fig. 3a, b), both DNA products of the expected size, 100 and 136 bp, respectively, were observed. The ability of P-1 to degrade crude oil, fractions A5 and P3 of crude oil, hexadecane and phenanthrene was determined by measuring the amount of residual hydrocarbons in the culture medium after 28 days of culture. Among the analyzed hydrocarbons crude oil, fractions A5 and P3 of crude oil and hexadecane were degraded with various efficiency, and no degradation of hydrocarbon was observed in the medium supplemented with phenanthrene (96 % of phenanthrene remained in the culture) as a sole carbon source. The highest degradation efficiency was observed in the culture supplemented with fraction A5 of crude oil (39 % of hydrocarbon was degraded at the end of the experiment). In the media supplemented with crude oil and fraction P3 of crude oil, 27 % of hydrocarbons were degraded and only 13 % of hexadecane was degraded in the medium with this hydrocarbon as the only carbon source (Fig. 4).

In order to check if P-1 produces biosurfactants on molasses, the surface-active properties of cell-free supernatant were determined (Table 1). The surface tension of the supernatant was reduced to 26.67 mN m^−1^, and the flat shape of the oil drop added to the surface of the culture supernatant, indicating a positive result in the drop-collapse method, was observed. Moreover, the clear zones generated in the oil-spreading test and the high emulsification activities of the strain P-1 were recorded. The concentration of the produced rhamnolipid was high and reached the value of 30.23 g l^−1^ (Table 1).

**Table 1:** Surface-active properties of molasses culture and concentration of rhamnolipid produced by P-1

Surface tension (mN × m^−1^ )	Drop-collapse	Oil-spreading (mm)	MBA	Emulsification index (%)			Rhamnolipid yield (g l^−1^)
				C	D	H	
26.67 ± 0.35	+	47.33 ± 1.15	+	68.8 ± 4.83	67.63 ± 2.26	70.1 ± 0.39	30.23 ± 2.97

± Standard deviation of three independent experiments

*MBA* methylene blue agar, *C* cyclohexane, *D* diesel oil, *H* hexadecane

**Fig 5:**
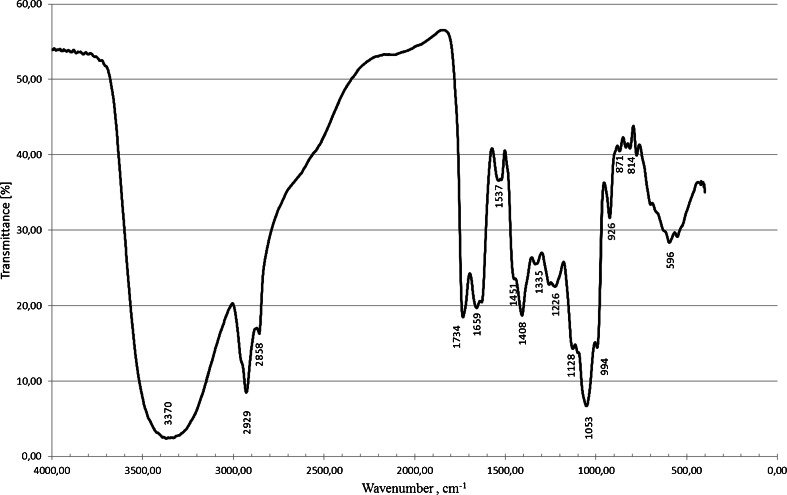
FT-IR spectrum of the crude biosurfactant produced by P-1 strain cultivated in molasses medium

The molecular composition of the biosurfactant produced by *Pseudomonas* sp. P-1 was evaluated by FT-IR. The obtained infrared spectrum disclosed a broad stretching peak at 3,370 cm^−1^, indicating the presence of –OH bonds (free hydroxyl groups of rhamnose rings). Absorption around 2,929 and 2,858 cm^−1^ is assigned to the symmetric stretch (–CH) of –CH_2_ and –CH_3_ groups of aliphatic chains. The absorption peak located at 1,734 cm^−1^ indicates the presence of ester carbonyl groups (–C = O bond) in biosurfactants. The ester carbonyl group from the bands at 1,128 cm^−1^ and C–O–C vibrations at 1,053 cm^−1^ (rhamnose rings) were also proved. Protein-related bands, the –C = O amide I (1,659 cm^−1^) and –NH/–C = O combination of the amide II bands (1,537 cm^−1^), were observed. It might be possible that the additional bands at 1,659 and 1,537 cm^−1^ resulted from polypeptide contamination resulting from cell debris co-precipitated with the biosurfactant during the extraction process. The absorption peak at 994 cm^−1^ may indicate the presence of polysaccharide or polysaccharide-like substances in the biosurfactants (Fig. 5)

**Fig. 6:**
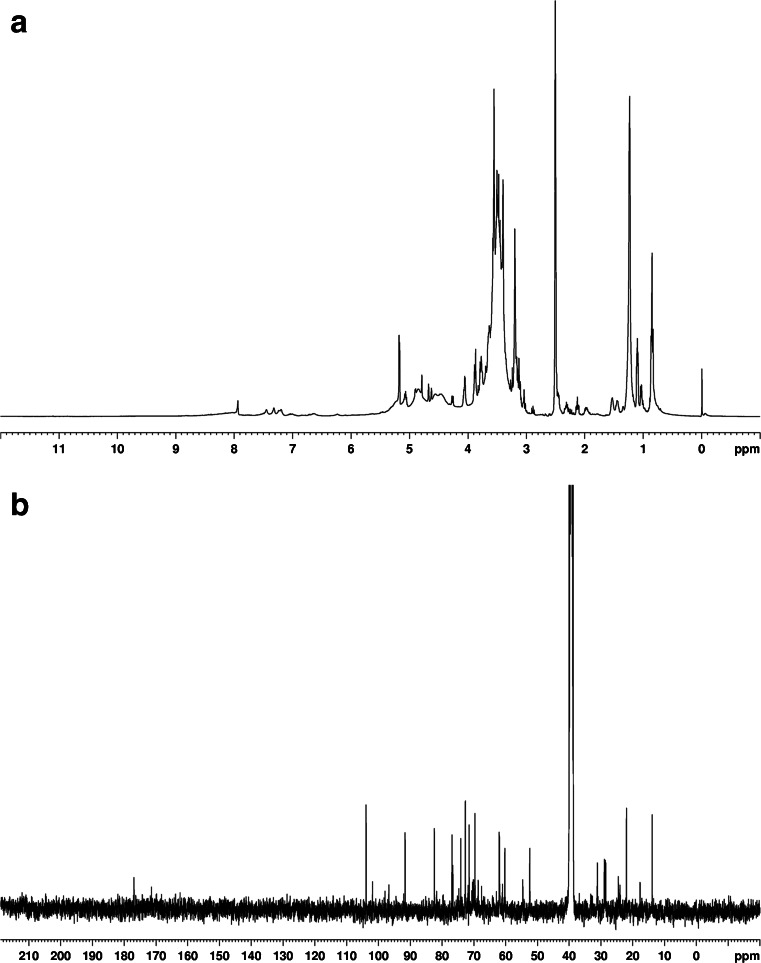
NMR spectra of crude biosurfactant produced by P-1 strain cultivated in molasses medium: **a** ^1^H NMR; **b** ^13^C NMR

The chemical structure of the rhamnolipids present in the analysed mixture of biosurfactants was confirmed by nuclear magnetic resonance spectroscopy. The obtained ^1^H NMR spectra strongly indicated that the brownish oily substance produced by P-1 was a mixture of glycolipids, and not another group of biosurfactants (Fig. 6a). In the ^13^C-NMR spectrum lipid, signals of CH_2_ from *δ* 21.9 to 31.1 and CH_3_ at *δ* 13.8, and ester and carboxylic signals at *δ* 171.5 and *δ* 176.95 were observed (Fig. 6b).

Up to 11 rhamnolipid homologues were determined by LC-MS analysis in the mixture produced by the P-1 strain. Table 2 presents a list of RL congeners found in the analysed sample of crude biosurfactant. Negative ESI-MS spectra showed the main pseudo-molecular ions at *m*/*z* 677 and *m*/*z* 622, which corresponds to the deprotonated molecules [M-H]^¯^ of the dirhamnolipids Rha-Rha-C_10_-C_12_ or Rha-Rha-C_12_-C_10_, and Rha-Rha-C_8_-C_10_, respectively. Minor ions at *m*/*z* 649 (Rha-Rha- C_10_-C_10_), *m*/*z* 475 (Rha-C_8_-C_10_ or Rha-C_10_-C_8_) and *m*/*z* 503 (Rha-C_10_-C_10_) were observed in the analysed mixture of biosurfactants. RL with some unsaturated fatty acid, Rha-C_10_-C_12:1_/Rha-C_12:1_-C_10_ (*m*/*z* 529), Rha-Rha-C_10_-C_12:1_ (*m*/*z* 675) and Rha-C_8:2_ (*m*/*z* 301) were also present in the mixture. In general, the predominance of dirhamnolipids compared to monorhamnolipids was observed. The most frequent were di-rhamno-di-lipidic (four detected) and mono-rhamno-di-lipidic (three detected). The relative abundance of [M-H]^−^ mono-rhamno-mono-lipidic, mono-rhamno-di-lipidic and di-rhamno-di-lipidic structures were 0.8, 31.8 and 67.4 %, respectively.

**Table 2:** The chemical composition of rhamnolipid mixture produced by *P. aeruginosa* P-1 culture identified by LC-ESI-MS

Rhamnolipid congener	Molecular formula	Molecular weight [g mol^−1^]	Pseudomolecular ion (*m/z*)	Ion fragments *m*/*z*	Retention time [min]	Relative abundance [%]
Rha-C_8:2_	C_14_H_22_O_7_	302	301	–	9.2	0.8
Rha-C_8_-C_10_/ Rha-C_10_-C_8_^*^	C_24_H_44_O_9_	476	475	311; 169	18.5	14.1
Rha- C_10_-C_10_	C_24_H_48_O_9_	504	503	339; 333; 169;163;119;103	12.1	6.8
Rha-C_10_-C_12:1_/ Rha-C_12:1_-C_10_	C_28_H_50_O_9_	530	529	365; 333; 169; 163;119;103	16.1	10.9
Rha-Rha-C_8_-C_10_	C_30_H_54_O_13_	622	621	452; 311;169	16.9	21.3
Rha-Rha-C_10_-C_10_	C_32_H_58_O_13_	650	649	479; 339; 311; 246; 169	11.5	15.7
Rha-Rha-C_10_-C_12:1_	C_34_H_60_O_13_	676	675	479; 311; 195; 169; 119; 103	10.4	5.6
Rha-Rha-C_10_-C_12_/ Rha-Rha-C_12_-C_10_	C_34_H_62_O_13_	678	677	507; 479; 311; 205;197; 169	15.2	24.8

Negative ESI-MS, [M-H]^−^ = pseudomolecular ions

## Discussion

In the present study of the characteristics of the *Pseudomonas* sp. P-1 strain, a potential tool for the enhanced bioremediation of petroleum-contaminated soil was described. Members of the genus *Pseudomonas*, due to the presence of a complex enzymatic system, show a wide variety of metabolic and physiological properties. They are also the most predominant group of microorganisms that degrade xenobiotic compounds. The phylogenetic analysis of P-1 revealed that close relatives of this strain are strains belonging to *P. aeruginosa.* It has been reported that various bacteria from genus *Pseudomonas*, including *P. aeruginosa* strains, inhabit oil-contaminated soils and can break down more than 100 different organic compounds (Glick et al. 1994; Hong et al. 2005; Saikia et al. 2012). They are the best known bacteria capable of utilizing a number of aliphatic and aromatic hydrocarbons as carbon and energy sources (Das and Chandran 2011; Kadali et al. 2012; Puškárová et al. 2013). Strain *Pseudomonas* sp. P-1 was characterized by its high removal of hydrocarbons contained in crude oil and its fractions A5 and P3. It exhibited high ability to utilize light boiling fraction (A5) of crude oil and had similar potential to degrade crude oil and its high boiling fraction (P3). The reason of lower degradability of the P3 was its molecular size and physico-chemical parameters (especially water solubility). The ability of bacteria to degrade hydrocarbons from fraction A5 with higher efficiency as compared to fraction P3 was reported by Płaza et al. (2008). The capability of other *Pseudomonas* strains to remove crude oil, kerosene and lubricant oil (Silva et al. 2006), and crude, diesel and engine oils (Obayori et al. 2009) was reported earlier for *P. aeruginosa* AT18 and *Pseudomonas* sp. LP1 strains, respectively. P-1 also exhibited the ability to degrade hexadecane, but did not use naphthalene as a carbon source. This was surprising because the presence of genes responsible for naphthalene degradation in the genome of P-1 was proved by the PCR method. This indicates that P-1 was only able to utilize aliphatic hydrocarbons, and the naphthalene dioxygenase *nahAc* possessed by this strain gene was disabled. The ability of *Pseudomonas* strains to degrade only one type of hydrocarbon was also observed by Kumar et al. (2006), who found that IR1 was capable of utilizing two-, three- and four-ring PAHs but not hexadecane and octadecane.

Strains belong to the genus *Pseudomonas* are among the best known biosurfactant producers. Since biological surface-active compounds are considered to be very useful in improving the bioavailability of hydrocarbon pollutants in soil, the ability of biosurfactant production in combination with the capacity to degrade hydrocarbons makes pseudomonads one of the most useful tools in bioremediation of petroleum-polluted soils. The ability of P-1 to synthesize biosurfactants was tested on a blood agar plate. The obtained positive result for the hemolytic activity of P-1 was insufficient to confirm about biosurfactant production by this strain. Since it is known that pseudomonads possess, apart from biosurfactants, β-hemolysins, which also cause clearing of the blood agar, the application of additional tests is required. The blue agar plate method, specially developed for the detection of glycolipid production such as rhamnolipids by *Pseudomonas* sp., was proposed by Siegmund and Wagner (1991). A new approach for the determination of the potential ability of bacterial strains for biosurfactant production is the detection of genes coding enzymes involved in their biosynthesis (Satpute et al. 2010). The polymerase chain reaction screening method is now a widely used technique. This approach was used for the detection of *Bacillus* strains with the potential to produce lipopeptide biosurfactants (Hsieh et al. 2004; Tapi et al. 2010), and is becoming an increasingly employed approach in the search for biosurfactant-producing pseudomonads. The use of methylene blue indicator plates and PCR of rhamnosyl transferase gene (*rhlAB*) confirmed the production of anionic glycolipid biosurfactant (rhamnolipid) by strain P-1. The usefulness of the CTAB–methylene blue agar test for the screening of a bacterial rhamnolipid producer was reported by Gunther IV et al. (2005). Kumar et al. (2008) used both of these methods to indicate rhamnolipid synthesis by *P. aeruginosa* DHT2. Similarly, Shoeb et al. (2012) applied the detection of the *rhlAB* gene through PCR for establishing the ability of eight bacterial strains from the genus *Pseudomonas* to produce the rhamnolipid type of biosurfactant.

Interest in biosurfactant applications in many industries and environmental protection has recently significantly increased. However, the success of their production depends on the increase of yield, the development of economical engineering processes and the use of low cost effective renewable agro-industrial substrates for their production. The search for inexpensive raw materials is important in the overall economy of biosurfactant production since they account for 10–30 % of the total cost (Cameotra and Makkar 1998). The main problem in the use of raw substrates is the selection of suitable waste materials with the right balance of nutrients adequate for cell growth and biosurfactant accumulation. It is known that pseudomonads are able to grow on numerous low-cost raw substrates, but often they do not synthesize sufficient amounts of biosurfactant (Makkar et al. 2011). Of 21 low-cost raw materials, only molasses appeared to be good material for P-1 growth and rhamnolipid production with high efficiency (Pacwa-Płociniczak et al, Evaluation the usefulness of *Pseudomonas aeruginosa* P-1 strain for bioremediation of petroleum-polluted soils, Personal Communication). A few attempts to use agro-industrial waste substrates for biosurfactant production by bacteria from the genus *Pseudomonas* have been made. It has been demonstrated that rhamnolipids can be produced using distillery and curd whey waste (Dubey and Juwarkar 2001), waste soybean soapstock (Nitschke et al. 2005), glycerol (Silva et al. 2010) oil, or waste coconut frying oil (George and Jayachandran 2012). However, in most cases, the rhamnolipid yield using low-cost substrates does not exceed 10 g l^−1^. Optimization of rhamnolipid production using waste materials by changing carbon and nitrogen sources, the ratio of carbon to nitrogen source, pH and temperature conditions, studied by Xia et al. (2012), enhanced the rhamnolipid yield to 50.2 g l^−1^. Nevertheless, all the individual modifications of fermentation conditions make the biosurfactant synthesis more expensive, therefore the application of raw, unchanged materials as substrates for high biosurfactant synthesis is preferred. Here, in our study, we confirmed the usefulness of molasses as a material for the fermentative production of rhamnolipid by the P-1 strain. The obtained rhamnolipid synthesis can be considered as very high (30.23 g of rhamnolipid per litre of molasses medium) compared to other strains for which rhamnolipid yield did not exceed 0.4 g l^−1^ (Onbasli and Aslim 2009).

A practical measure of the utility of a biosurfactant solution or biosurfactant-producing strain is its ability to emulsify non-aqueous liquids (Ghurye et al. 1994).

The emulsification activity of the cell-free molasses was 68.8, 67.6 and 70.1 % with cyclohexane, diesel oil and hexadecane, respectively. In similar investigations, *Pseudomonas* biosurfactant producers were able to emulsify hydrocarbons with similar efficiency (Rahman et al. 2002; Kumar et al. 2008; Aparna et al. 2012). The obtained results suggested that the application of this cell-free molasses supernatant in the bioremediation process may enhance the availability of the hydrocarbons in soil.

The spectroscopic analysis of crude biosurfactant produced by P-1 cultivated in molasses medium confirmed that it was a mixture of glycolipids, and not another group of biosurfactants. This mixture consisted of different rhamnolipidic congeners (mono-rhamno- and di-rhamno-di-lipidic). The di-rhamno-di-lipidic congeners showed the highest relative abundance of [M-H]. It has been reported that the composition and predominance of a particular type of congener of rhamnolipids produced by pseudomonads often depend on the type of used carbon source (Déziel et al. 1999). Similar to our results, a higher proportion of dirhamnolipid compared to monorhamnolipid was obtained when *Pseudomonas* strains were cultivated using soybean oil (Rahman et al. 2002) and modified peptone glucose ammonium salt (PPGAS) containing 1 % of molasses (Aparna et al. 2012). However, the predomination of monorhamnolipids was observed when the *Pseudomonas* strain was grown in media composed of residues from soybean, corn, babassu, cottonseed and palm oil refineries (Nitschke et al. 2005).

## Conclusions

The results of this study indicate that the *Pseudomonas* sp. P-1 strain has the ability to degrade various hydrocarbons (hexadecane, crude oil and fractions A5 and P3 of crude oil), and is efficient in rhamnolipid production and hydrocarbon emulsification. Our study confirmed that molasses can serve as an efficient and low-cost medium for bacterial culturing and synthesis of rhamnolipid by P-1. This finding has a practical advantage because the use of low-cost raw materials as a substrate for P-1 growth and biosurfactant production may result in wider application of this strain in bioremediation. The features of the P-1 strain make it promising agent for cleaning up environments contaminated with petroleum compounds. Because the potential of bacteria for bioremediation application is highly dependent on biotic and abiotic soil parameters, further studies to check its activity and ability to survive in hydrocarbon-contaminated soil will be carried out.
